# GLP-1 Receptor Agonists for Obesity Management in Older Adults: A Scoping Review on the Risk of Sarcopenia and Sarcopenic Obesity

**DOI:** 10.1007/s13668-026-00777-x

**Published:** 2026-06-17

**Authors:** Hilal Simsek, Asli Ucar

**Affiliations:** 1 Department of Nutrition and Dietetics, Bor Faculty of Health Sciences, Nigde Ömer Halisdemir University, Nigde, Türkiye; 2 Department of Nutrition and Dietetics, Faculty of Health Sciences, Ankara University, Ankara, Türkiye

**Keywords:** Anti-obesity medications, Glucagon-like peptide receptor agonists (GLP-1RAs), Sarcopenia, Sarcopenic obesity

## Abstract

**Purpose of Review:**

Obesity pharmacotherapy has become increasingly relevant in older adults due to challenges in lifestyle modification and the burden of non-communicable diseases. Despite the expanding use of anti-obesity medications (AOMs), sarcopenia and sarcopenic obesity (SO) remain major concerns in this population. This review synthesizes current evidence on glucagon-like peptide-1 receptor agonists (GLP-1 RAs), a well-studied AOMs, focusing on their effects on sarcopenia through mechanistic insights and clinical considerations in older adults.

**Recent Findings:**

GLP-1 RAs effectively induce weight loss; however, concomitant muscle mass loss is common, raising concerns about sarcopenia. Weight cycling introduces uncertainty regarding skeletal muscle health and SO risk. Although data on muscle mass indices and physical performance remain limited and inconsistent, evidence suggests favorable effects on muscle metabolism and composition. Mechanistically, these effects are linked to GLP-1 RA-mediated suppression of inflammatory pathways, modulation of skeletal muscle metabolism, and preservation of mitochondrial function, thereby influencing insulin resistance. The most common adverse events are mild to moderate gastrointestinal symptoms, which may be mitigated through dietary counseling during dose escalation.

**Summary:**

Integrating GLP-1 RA therapy with tailored resistance exercise and caloric restriction with dietary counseling, including adequate protein intake (1.2–1.6 g/kg/day), may help preserve muscle mass and function in older adults. Implementation of GLP-1 RA-specific medical nutrition therapy protocols, with dietitian involvement to optimize dietary recommendations and manage adverse events, is recommended. Studies addressing skeletal muscle quantity, quality, and functional capacity are needed to clarify the effects of GLP-1 RAs on sarcopenic obesity in older adults.

## Introduction

The combined impact of increasing global life expectancy and the obesity epidemic poses a significant threat to public health and the stability of healthcare systems due to the rising costs of treating obesity and its associated comorbidities [1]. Sarcopenia, one of the most significant geriatric syndromes given its central role in maintaining functional independence, also constitutes a critical element at this intersection. Sarcopenic obesity (SO) is defined as the coexistence of excess adiposity combined with loss of muscle mass and function, representing a significant public health issue in the geriatric population [2].

Although prevalence estimates vary according to diagnostic criteria, the global prevalence of SO among adults aged 60 years and older has been reported 11% [3]. SO is projected to affect up to 200 million individuals over the next 35 years, underscoring its potential as an increasingly important public health issue [4]. Indeed, the presence of sarcopenia and obesity, as well as SO with its dual impact, contributes substantially to national and global healthcare expenditures and serves as a prognostic factor for the growing burden on public health [5, 6]. Previous studies have estimated that the economic burden of sarcopenia in the United States accounts for approximately 1.5% of total national health expenditures [7], with annual per capita healthcare costs rising by $2,315.7 in individuals with sarcopenia [8]. Obesity, primarily associated with the increased burden of non-communicable diseases (NCDs), has been linked to healthcare costs approximately 30% higher than those of individuals with normal body weight [9]. Given the higher risk associated with SO for functional capacity decline, cardiovascular health, and all-cause mortality, it is anticipated that SO may result in more severe public health consequences than obesity alone [10].

In the context of an aging population and its associated public health challenges, addressing obesity in older adults is particularly demanding due to barriers such as resistance to lifestyle changes. Within this framework, anti-obesity medications (AOMs) emerge as a supportive strategy under pharmacotherapy, which is one of the three main evidence-based approaches to obesity treatment [11, 12]. Despite the common use of pharmacotherapy in obesity management, sarcopenia and SO remain significant concerns [12]. This review examines the body of evidence for GLP-1 receptor agonists (GLP-1RAs), a class of AOMs relatively frequently used in older adults, focusing on sarcopenia through mechanistic insights and clinical considerations.

## The Dual Challenge: Obesity and Sarcopenia in Older Age

Age-related changes in body composition are characterized by decreased fat-free mass (FFM), which includes reductions in total muscle mass and bone mineral density (BMD), alongside a relative increase in fat mass (FM) [13]. These changes, which are critical for the maintenance of functional capacity, are mediated by a complex array of metabolic and biochemical processes, including altered muscle metabolism related to declining anabolic hormones with age, elevated myostatin levels, changes in myokine and adipokine profiles, and inflammation [4]. Increased adiposity, which plays a central role in both aging and obesity-related pathologies, is responsible for enhanced oxidative stress and chronic low-grade inflammatory responses by promoting free radical production and the release of pro-inflammatory cytokines. Oxidative stress and chronic low-grade inflammation, in turn, exacerbate muscle catabolism and negatively affect functional capacity [4, 14].

The pathophysiology of obesity and sarcopenia is considered a multifaceted vicious cycle rather than a simple causal relationship [4]. Indeed, age-related declines in skeletal muscle mass index (SMI) and function often occur in parallel with increases in total FM, while the clinical course of obesity is characterized by a range of risk factors for sarcopenia, including elevated oxidative stress, inflammation, and the burden of NCDs [15, 16].

### Pharmacotherapy for the Management of Obesity in Older Adults & GLP-1 Receptor Agonists

Evidence-based obesity treatment is classified into three main approaches: lifestyle interventions encompassing behavioral modification, diet, and physical activity; bariatric surgery; and pharmacotherapy. In comprehensive obesity management, patient-centered strategies incorporating different combinations of these interventions within individualized obesity care plans are recommended [17–19]. However, a detailed discussion of comprehensive obesity management is beyond the scope of this review. Instead, this paper focuses on Glucagon-Like Peptide-1 receptor agonists (GLP-1 RAs) as commonly used anti-obesity medications (AOMs) and summarizes the current evidence on their efficacy and safety, particularly from the perspective of sarcopenia in older adults.

Pharmacotherapy has emerged in recent years as a promising strategy for the management of obesity and its associated comorbidities. In older adults, lifestyle modifications remain a significant challenge [11, 20], and the increasing burden of NCDs further underscores the strategic role of pharmacotherapy as part of a comprehensive strategy of the obesity management. However, evidence regarding its use in individuals aged 65 years and older remains limited [12]. This highlights the need for careful evaluation of the efficacy and safety profile of AOMs in the older population. Currently, seven AOMs are approved by both the Food and Drug Administration (FDA) and European Medicines Agency (EMA) -liraglutide, semaglutide, tirzepatide, orlistat, naltrexone/bupropion, setmelanotide, and metreleptin- while two additional agents (phentermine and phentermine/topiramate) are FDA-approved only. Ongoing research continues to evaluate the efficacy and safety of these and other emerging therapies [21, 22].

GLP-1 receptor agonists (GLP-1 RAs), which regulate glucose metabolism through mechanisms such as enhancing glucose-dependent insulin secretion via the incretin effect, suppressing glucagon secretion, delaying gastric emptying, and supporting pancreatic β-cell proliferation, are primarily used in the management of type 2 diabetes mellitus (T2DM) and, in certain cases, obesity [23]. Since their initial approval in the early 2000 s, the pharmacotherapy of GLP-1 RAs has been well recognized not only for achieving glycemic targets but also for their effectiveness in promoting satiety and weight loss, as well as their association with reductions in cardiovascular risk and all-cause mortality; current research is also exploring novel GLP-1 RA combinations [23, 24]. The most widely used GLP-1 RAs today and those currently attributed with the most clinical evidence in weight management include Liraglutide, Semaglutide, and Tirzepatide (as a dual agonist of GLP-1 and glucose-dependent insulinotropic polypeptide) [25]. In addition to these widely used AOMs, Exenatide, one of the earliest GLP-1 RAs approved by the FDA and EMA for T2DM management, is not indicated for weight management but remains clinically relevant, particularly in older adults with comorbid obesity and T2DM, given its long-term clinical experience and established tolerability profile [26–28].

### Therapeutic Indications and Dosing Protocols

Among GLP-1 receptor agonists approved for weight management, liraglutide (SAXENDA) is one of the earliest agents to receive authorization from both the FDA and EMA. It is indicated for patients with obesity (BMI ≥ 30 kg/m^2^) or overweight (BMI 27–29.9 kg/m^2^) in the presence of at least one weight-related comorbidity [29, 30]. Semaglutide is another GLP-1 RA used in the pharmacological management of T2DM and/or obesity. Currently, both the subcutaneous injectable form (OZEMPIC) and the oral formulation (RYBELSUS) are approved by the FDA and EMA for use in T2DM as an adjunct to diet and exercise. Another subcutaneous injectable version (WEGOVY) is approved for the same indication of obesity or overweight with at least one weight-related comorbidity, in combination with diet and exercise [31–33].

Tirzepatide is a dual agonist at GLP-1 and glucose-dependent insulinotropic polypeptide (GIP) receptors and produces enhanced glycemic control and greater weight loss compared to many GLP-1 RAs [34]. Tirzepatide (MOUNJARO) is a once-weekly subcutaneous injectable agent first authorized for T2DM and later extended to the management of obesity or overweight with at least one weight-related comorbidity, thereby aligning its therapeutic scope with subcutaneous liraglutide and semaglutide [34–36].

The therapeutic regimen of GLP-1RAs for weight management involves a gradual dose escalation protocol to optimize tolerability and clinical response. The therapeutic regimen of liraglutide involves gradual titration to a target subcutaneous dose of 3 mg/day, and its safety and efficacy profiles in older adults parallel those observed in younger adults; therefore, no age-based dose adjustment is recommended [29, 37, 38]. Similarly, semaglutide for weight loss is titrated to a target subcutaneous dose of 2.4 mg/week, while its oral formulation -approved for T2DM management- is administered at 3–14 mg/day [39]. As with liraglutide, semaglutide demonstrates comparable treatment outcomes across age groups, and routine dose modification in older adults is not required [12, 33]. Tirzepatide also employs a gradual dose escalation, starting at a subcutaneous dose 2.5 mg/week and increasing up to 15 mg/week, with no routine age-based dose adjustment required [36, 39, 40].

### Evidence from Clinical Trials on Weight Loss and Body Composition

Liraglutide-based clinical evidence from the SCALE (Satiety and Clinical Adiposity-Liraglutide Evidence) trials (NCT01272219 and NCT01272232), which included older adults within the adult study population, reported that a 56-week intervention with liraglutide 3 mg/day as an adjunct to lifestyle modification resulted in significant weight loss compared with placebo [41, 42]. These effects were consistent across participants aged ≥ 65 and < 65 years [12, 43]. Similarly, in a recent retrospective cohort study by Oral et al., 32 patients aged > 60 years with obesity or overweight and at least one weight-related comorbidity experienced a 5–10% reduction in baseline body weight following 24 weeks of liraglutide treatment, although body composition was not assessed in this study [44]. Evidence regarding the effects of liraglutide on body composition primarily comes from adult populations that did not include older adults; across 24-to-40-week interventions, reductions in fat mass and/or lean mass have been reported [45–47], alongside improvements in thigh muscle fat and muscle volume [48]. A recent systematic review indicated that while reductions in fat mass and visceral adipose tissue are generally consistent in obese or overweight adults, changes in lean mass are variable, showing decreases, preservation, or increases [49]. In older adults, the effects of liraglutide on body composition remain insufficiently characterized; within this context, a case series by Perna et al. involving sedentary overweight and obese older adults with T2DM (*n* = 9) demonstrated reduced fat mass and improved SMI after 24 weeks of treatment [50].

Semaglutide-based clinical evidence from a post hoc analysis of the SUSTAIN (Semaglutide Unabated Sustainability in Treatment of Type 2 Diabetes) trials (SUSTAIN 1–5; NCT02054897, NCT01930188, NCT01885208, NCT02128932, NCT02305381)-lasting between 30 and 56 weeks and focusing on participants aged ≥ 65 years- showed that once-weekly injectable semaglutide produced dose-dependent reductions in body weight, with slightly greater weight loss observed in older adults compared with those < 65 years. Specifically, weekly doses of 0.5 mg and 1.0 mg resulted in mean weight reductions of −3.6 to −4.6 kg and −4.1 to −6.7 kg, respectively, in the older population [51]. However, body composition changes were not evaluated in this analysis [51], and data on fat or lean mass remain limited. Similarly, a retrospective cohort study in T2DM patients treated with subcutaneous semaglutide reported similar weight loss at 3 and 6 months in older (> 65 years) and younger (< 65 years) adults, but muscle mass, strength, and other body composition parameters were not measured, preventing conclusions about effects on sarcopenia [52]. Notably, the STEP (Semaglutide Treatment Effect in People with obesity) trials, which specifically examined body composition, demonstrated substantial weight loss compared with placebo (NCT03548935) [53], and reductions in fat mass and visceral adipose tissue were accompanied by decreases in lean mass, although the proportion of lean mass relative to total body mass increased (NCT04074161) [54]. Importantly, a pilot study in older adults with T2DM and obesity reported that semaglutide preserved the SMM percentage of the extremities despite a decrease in total muscle mass [55]. Similarly, Volpe et al. observed that in T2DM patients (mean age: 64.9 years) treated with subcutaneous semaglutide, reductions in the SMI were smaller than those in the fat mass index (FMI) [56], and the findings suggest a preservation of muscle strength and muscle quality.

Data from the PIONEER (Peptide Innovation for Oral Delivery of Semaglutide in Type 2 Diabetes) trials (NCT03018028 and NCT03015220) indicated that oral semaglutide also led to dose-dependent weight loss in both younger and older adult groups, but body composition was not assessed [57]. Another study evaluating the 24-week efficacy of oral semaglutide in 25 patients with T2DM aged 20–78 years reported preservation of lean mass and appendicular skeletal muscle mass index (ASMI), along with reductions in fat mass. Although no subgroup analysis was performed specifically for older adults, changes at the end of 24 weeks were reported to be independent of age [58].

Conversely, semaglutide-focused studies in older adults have accelerated in recent years and appear to be highly promising [59–61]. Among these, the 24-month retrospective study by Ren et al. is particularly important because it directly examines semaglutide use in older adults with T2DM with an explicit focus on sarcopenia and provides relatively long-term outcomes [60]. According to this study, semaglutide use was associated with reductions in muscle mass and ASMI; while handgrip strength initially increased in men, it later declined in both sexes, and walking speed significantly decreased in both men and women. Notably, higher semaglutide dosage (mg) emerged as an independent predictor of these adverse musculoskeletal changes. [60].

Tirzepatide-based evidence from the SURPASS (Researching Tirzepatide in Patients with Type 2 Diabetes for Glycemic Control and Weight Management) trials (SURPASS-1 NCT03954834 and SURPASS-5 NCT04039503) demonstrated dose-dependent, significant weight loss over 40 weeks compared with placebo, along with reductions in BMI and waist circumference [62, 63]. Pooled post-hoc analyses of SURPASS 1–5 (NCT03954834, NCT03987919, NCT03882970, NCT03730662, and NCT04039503) showed that weight loss was similar in participants aged ≥ 60 and < 60 years [64]; however, this population was not evaluated in terms of detailed body composition, which is particularly relevant in older adults. A recent post-hoc analysis of SURPASS-3 (NCT03882970) in adults with T2DM reported decreases in fat mass and muscle fat infiltration (MFI) accompanying weight loss [65], suggesting potential improvements in muscle quality that may be especially promising for older adults.

As a key trial evaluating the efficacy of tirzepatide, SURMOUNT-1 (NCT04184622) was consistent with the SURPASS studies in demonstrating dose-dependent weight loss [66]. Additionally, detailed body composition analyses were conducted in subgroups of this trial. At the end of 72 weeks, the change in the ratio of total fat mass to lean mass was 0.24 for tirzepatide versus 0.06 for placebo, indicating a greater improvement with tirzepatide [67]. A post-hoc age subgroup analysis of SURMOUNT‑1 further showed that tirzepatide reduced fat mass by 33–36% and lean mass by 10–11% across all age groups, resulting in improved body composition. Importantly, older adults (≥ 65 years) experienced similar changes without evidence of excessive lean mass loss [67]. These findings are further supported by another sub-analysis of SURMOUNT‑1, which reported a 33.9% (8.2% for placebo) reduction in fat mass and a 10.9% (2.6% for placebo) reduction in lean mass by week 72, with approximately 75% of weight loss attributable to fat mass, consistent across clinically relevant subgroups [68]. Also, a recent systematic review indicates that tirzepatide treatment in adults is associated with reductions in fat mass, relative preservation of lean mass, and decreases in MFI [69], all of which are critical for sarcopenia risk and functional capacity. These effects suggest potential improvements in muscle quality and composition, which may be particularly relevant for older adults at risk of muscle loss, although studies specifically focused on this population remain limited.

Although liraglutide, semaglutide, and tirzepatide have become the predominant GLP-1 RAs for obesity management, exenatide remains a clinically relevant option, particularly for older adults managing both obesity and T2DM [70]. Clinical evidence indicates that exenatide effectively reduces body weight and enhances metabolic control, with subgroup analyses confirming that these benefits are also observed in older adults [26, 71, 72]. Additionally, evidence from a meta-analysis confirmed its effectiveness in promoting weight loss among individuals with overweight or obesity without diabetes [73]. Notably, concerning body composition and muscle health, existing evidence suggests that weight loss associated with exenatide is primarily attributable to reductions in fat mass, whereas lean body mass is largely preserved [74]. However, body composition data compared to other GLP-1 RAs remain limited.

### Safety and Clinical Management

Safety trials of GLP-1 RAs, including liraglutide, tirzepatide, and semaglutide (oral or subcutaneous), indicate that the most common adverse events (AEs) of treatment are mild to moderate gastrointestinal including nausea, vomiting, diarrhea, constipation, dyspepsia, and decreased appetite. AEs are more frequent in older adults compared to young adults, and they are particularly noted to occur during dose escalation [12, 43, 57, 75].

Although reported AEs are generally not serious and safety and efficacy in older adults are generally comparable to those in younger adults [36], higher AE rates may nonetheless predispose older adults to additional risks that are more critical in this population, such as hypoglycemia, dehydration, or impaired renal function. Furthermore, in the context of polypharmacy, gastrointestinal side effects may have heightened clinical relevance, underscoring the importance of patient-based risk assessment in clinical practice. [75]. It should also be noted that these trials predominantly included relatively healthy older participants, as individuals with potential comorbidities such as cardiovascular or renal diseases were often excluded [12, 34].

Another important issue that makes AE management critical in older adults is the higher rate of treatment discontinuation. Although discontinuation due to AEs does not always correlate directly with dose escalation [66], higher discontinuation rates have been reported in older adults [76]. To achieve optimal clinical outcomes, these AEs and their associated health risks underscore the necessity of patient-based monitoring, which should be carefully implemented [33, 51].

## Mechanistic Insights for Glp-1 Receptor Agonists and Risk of Sarcopenia

**Fig. 1:**
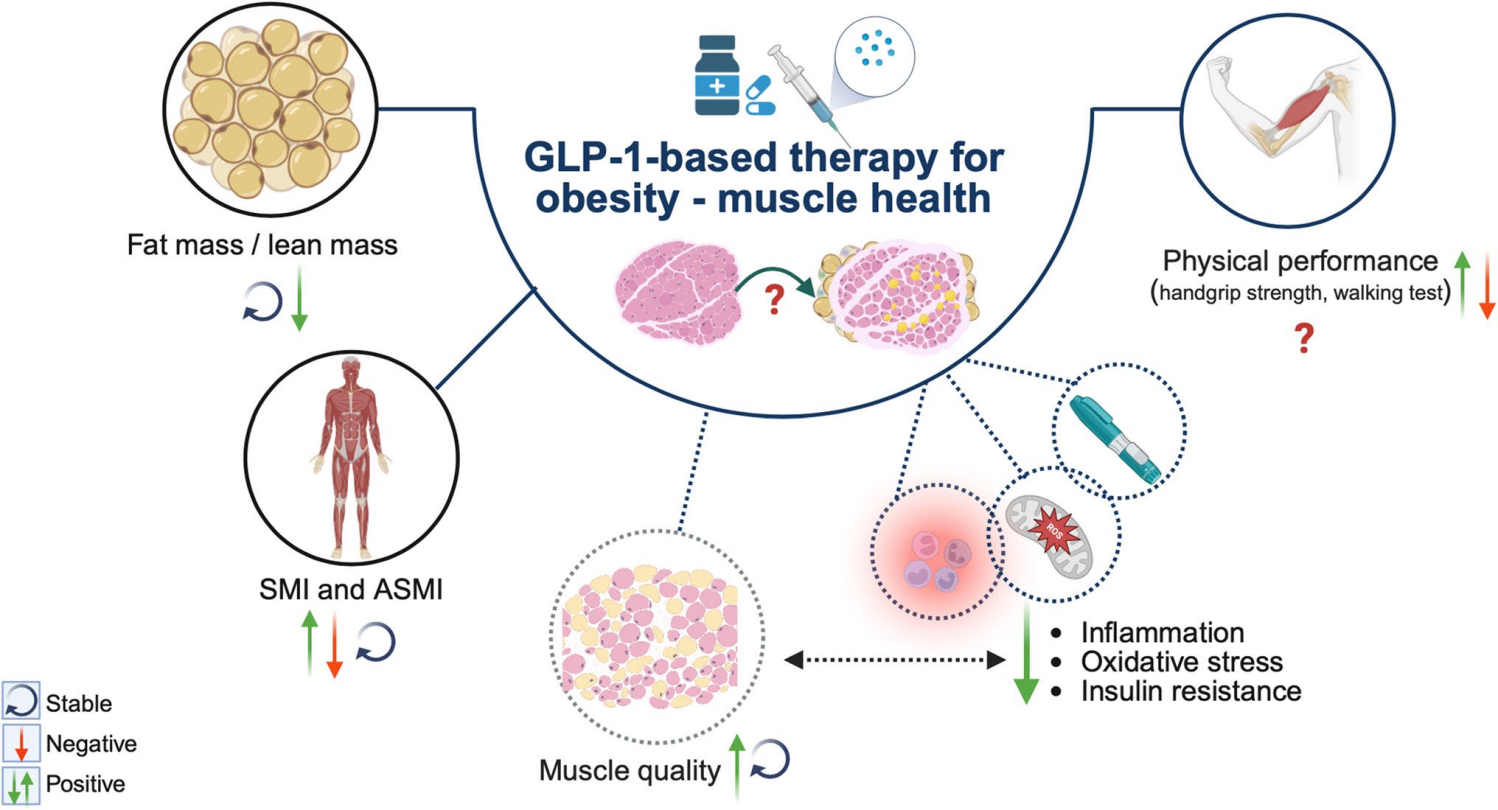
Main mechanisms for GLP-1 RAs and risk of sarcopenia. Abbreviations: SMI, skeletal muscle mass index; ASMI, appendicular skeletal muscle mass index; GLP-1, Glucagon-Like Peptide-1. This figure is illustrated with BioRender.com

Although GLP-1 RAs primarily induce weight loss through reductions in fat mass, they may also lead to a meaningful decrease in lean mass; indeed, a recent meta-analysis reported that approximately 25% of total weight loss is attributable to lean mass [77]. Furthermore, a network meta-analysis examining the effects of GLP-1 RA therapy on body composition reported a greater reduction in lean body mass in the intervention group compared with placebo or active comparators (mean difference = −0.86 kg, 95%CI: −1.30, −0.42). Changes relative to baseline lean mass were similar between the groups, and meta-regression analysis indicated that reductions in total body weight were significantly associated with fat mass loss but not with lean mass. Notably, the same meta-analysis highlighted that liraglutide, particularly when compared to higher doses of semaglutide or tirzepatide, promoted weight loss without a clinically meaningful reduction in lean mass [77]. In a meta-analysis in T2DM patients, pooled effects analysis demonstrated that GLP-1RAs were associated with a significant improvement in 6-min walk test results (mean difference = 22.3 m, 95% CI:1.64, 42.99) [78]. In older adults, the preservation of functional capacity and independence, as well as the prevention of frailty and sarcopenia, depends critically on maintaining adequate muscle (especially skeletal) mass and quality; consequently, this may represent a potential concern during GLP-1 RA-based obesity interventions. This section reviews alterations in muscle mass, muscle quality, and their underlying mechanisms during GLP-1 RA therapy, as illustrated in Fig. 1.

### Muscle Composition and Quality

Myosteatosis, characterized by abnormal fat infiltration in skeletal muscle (muscle fat infiltration, MFI) encompassing both intermuscular and intramyocellular fat, negatively affects muscle capacity and quality, leading to functional impairment and increased metabolic and physical health risks [79]. In this phenomenon, intramyocellular fat, which accounts for approximately 1.5% in healthy individuals, can rise to 5% in obesity and up to 11% with aging [80]. Intermuscular adipose tissue-derived inflammatory adipokines, exacerbated by aging, obesity, and sedentary behavior, promote both fat accumulation and chronic inflammation within skeletal muscle, driving adipogenic differentiation at the expense of myogenesis and thereby contributing to reduced muscle quality and increased metabolic risk [81].

Myosteatosis alters muscle fiber composition and organization, with a decrease in oxidative, insulin-sensitive fibers and a concomitant increase in relatively fast-fatiguing glycolytic fibers, paralleling the effects observed in obesity and aging. These alterations contribute to mitochondrial dysfunction, insulin resistance (IR), and the pathogenesis of sarcopenia [39, 81]. Given these pathophysiological changes, there has been growing interest in the potential therapeutic effects of GLP-1 RAs on MFI, inflammation, and muscle fiber characteristics [77, 82]. Indeed, GLP-1 RAs may support the preservation of muscle mass and function through improvements in glycemic control, weight reduction, and indirect modulation of inflammatory responses [81].

GLP-1RAs promote glucose uptake in the skeletal muscle by increasing microvascular blood flow and tissue perfusion and inhibit muscle catabolism via the AMP-activated protein kinase pathway [39, 77, 83]. Additionally, the increase in microvascular blood flow in muscles contributes to reduced intramuscular fat accumulation and alleviation of myosteatosis, while AMPK pathway-mediated increased fat oxidation supports the mobilization of intramuscular fat, allowing for improvements in energy metabolism and reduction of fat within muscle tissue [39]. At the proteolytic level, GLP-1 RAs may reduce muscle protease activity and modulate inflammatory pathways, thereby attenuating protein breakdown and contributing to muscle mass preservation [39, 84]. These complementary effects on fat and muscle catabolism suggest that GLP-1 RAs may help preserve skeletal muscle integrity while concurrently improving metabolic efficiency in the context of obesity-related muscle dysfunction.

Clinical trials investigating the effects of different GLP-1RAs on myosteatosis through magnetic resonance imaging (MRI) subgroup analyses in mostly middle-aged adults (mean ages 50 to 56 years) have generally reported promising findings. According to the post-hoc analysis of SURPASS-3 (NCT03882970), 52 weeks of tirzepatide treatment resulted in a 4.4% reduction in MFI in individuals with T2DM [85]. In individuals with obesity who do not have T2DM, Pandey et al. demonstrated that liraglutide reduced thigh MFI by 7.8%, suggesting an improvement in muscle composition [48]. In two studies examining the 6-month effects of semaglutide, patients with metabolic dysfunction-associated steatotic liver diseases (MASLD) and showed reduced skeletal muscle steatosis with preservation of muscle mass [86], whereas in patients with MASLD and human immunodeficiency virus, semaglutide led to a 9.3% decrease in psoas muscle volume with no change in muscle fat fraction [87]. Although findings from clinical studies are promising, there are concerns that long-term exposure of GLP-1 RAs at supratherapeutic or maximum approved maintenance doses may impair muscle cell differentiation and potentially contribute to the development of sarcopenia, despite their potential to improve muscle metabolism and quality [83, 88]. Furthermore, additional research is warranted to elucidate the effects of these agents on muscle fibers and tendons, which are critical for muscle function [83].

### Endocrine Properties, Oxidative Stress and Inflammation

Beyond its role as a primary regulator of movement and energy balance, skeletal muscle also has endocrine properties. Changes in muscle metabolism are frequently intertwined with mechanisms of oxidative stress, mitochondrial function, and inflammation. For instance, myosteatosis impairs muscle health through both metabolic dysfunction and inflammation, while chronic low-grade inflammation negatively affects metabolic balance by inhibiting protein synthesis and increasing proteolysis via signaling pathways [39]. Myokines produced by skeletal muscle, such as interleukin-6 (IL-6) and irisin, play a key role in mediating these immune and metabolic responses through autocrine, paracrine, and endocrine effects. Specifically, these two myokines are considered protective proteins released from muscle in response to exercise and are particularly important in responding to oxidative stress and maintaining mitochondrial function [39, 89]. Furthermore, based on preclinical evidence suggesting that muscle-derived IL-6 may enhance postprandial GLP-1 secretion [90], it has been proposed that GLP-1RAs could modulate metabolic and inflammatory processes through this pathway [39].

GLP-1 RAs exert immunomodulatory properties through peripheral and central pathways. Demonstrating peripheral effects by suppressing T-cell-mediated inflammation via GLP-1 receptor activation in intestinal lymphocytes, and central effects by mediating Toll-like receptor (TLR)-induced anti-inflammatory responses through GLP-1 receptor signaling in the brain [39]. Additionally, the suppression of pro-inflammatory cytokines (e.g., TNF-α, IL-6) and inflammatory pathways, including the blocking of NF-κB activation [39, 91], contributes to the establishment of an anti-inflammatory environment that can mitigate oxidative stress and inflammation induced muscle protein catabolism, thereby supporting the preservation of SMM and functional capacity [40].

Insulin resistance, a pathology closely associated with obesity, sarcopenia, and chronic low-grade inflammation -including age-related “inflammaging”- represents another key target of GLP-1RA actions. GLP-1 directly regulates insulin secretion, while GLP-1 RAs have the potential to improve insulin sensitivity and glucose homeostasis by attenuating inflammatory pathways such as NF-κB and the NLRP3 inflammasome signaling, thereby mitigating IR [40]. This mechanism converges with the previously detailed inflammatory mechanisms, particularly those mediated by pro-inflammatory cytokines, and the increase in adiposity, representing a shared pathological link between metabolic dysfunction and skeletal muscle impairment. GLP-1RAs may improve these metabolic outcomes while modulating inflammatory pathways that contribute to muscle loss and the development of sarcopenia.

### Interplay between GLP-1 Signaling and Muscle Metabolism

GLP-1RAs modulate metabolism through multiple intracellular signaling pathways, including cAMP/PKA, PI3K/Akt, and SIRT1-mediated cascades, influencing glucose uptake, mitochondrial function, and inflammatory responses [82], which may contribute to interactions between GLP-1RA therapy and skeletal muscle metabolism, potentially affecting muscle metabolism and quality.

Notably, Huang et al. reported elevated GLP-1 levels in individuals with sarcopenia, and in vitro analyses further suggest that chronic and high exposure to GLP-1 may impair normal muscle cell differentiation and function by disrupting intracellular energy transporter (GLUT-4) translocation and mitochondrial function [88]. Accordingly, these findings indicate that, in certain contexts, increased GLP-1 can negatively affect myogenic differentiation and muscle metabolic performance [83].

Nevertheless, several mouse studies indicate that GLP-1RAs can modulate skeletal muscle metabolism by attenuating obesity-induced muscle atrophy markers via GLP-1 receptor-mediated signaling pathways. These effects appear to be mediated, at least in part, through the GLP-1/Sirtuin (SIRT1) pathway or via suppression of myostatin, while concomitantly reducing inflammation, thereby mitigating muscle wasting associated with SO [92–94]. Although these preclinical findings are promising for muscle quality, they need to be confirmed in clinical studies, as human data remain scarce.

## Clinical Considerations and Management

Management of GLP-1 RA-based obesity treatment in older adults requires a holistic approach that addresses the risk of frailty, malnutrition, and sarcopenia. During gradual dose escalation, dietary strategies—such as encouraging slower eating, consuming smaller meals, and avoiding high-fat foods—are the primary steps for managing nausea, with anti-emetic therapy reserved only for when strictly needed [40, 95]. Furthermore, as diarrhea and constipation are among the most frequently reported gastrointestinal AEs stemming from altered intestinal motility, their management should be expanded to include dietary fiber adjustments and targeted fluid repletion. Patients should also be advised that while most of these symptoms diminish over time, constipation may persist and require ongoing nutritional management [40, 95]. In addition, close monitoring and patient-centered evaluation are essential for optimizing clinical outcomes [40].

While the loss of muscle mass accompanying weight reduction remains a key concern -particularly in older adults- findings regarding muscle quality are variable. Although individuals with obesity often exhibit greater muscle mass compared to those with normal body weight, their muscle quality is generally lower due to factors such as relative weakness and reduced mobility [15, 96]. Notably, caloric restriction (CR) and moderate weight loss (approximately 5–10%) improve insulin sensitivity despite the accompanying loss of muscle mass, and the observed reduction appears to result primarily from increased proteolysis rather than reduced protein synthesis. Improved insulin sensitivity may, in turn, suppress proteolysis through its anabolic actions. However, components of obesity pathology -particularly IR and low-grade inflammation- may impair these protective mechanisms, thereby contributing to the development of SO. Furthermore, GLP-1-RAs may influence this mechanism indirectly by enhancing insulin sensitivity and exerting anti-inflammatory effects [84, 96]. Taken together, it has been proposed that muscle loss during GLP-1 RAs-based therapy is largely adaptive and proportional to overall weight reduction, and that improvements in muscle quality and insulin sensitivity may help offset potential functional decline [97].

Pharmacological treatment of obesity is associated with post-cessation weight regain or weight cycling a further concern in the context of sarcopenia. Although GLP-1 RA therapy has shown promising results for preserving lean mass and even muscle quality compared to fat mass, the trajectory of muscle mass and function following weight regain remains uncertain [98]. Moreover, weight cycling has previously been associated with lower muscle mass and strength, as well as an increased risk of SO [99, 100].

**Fig. 2:**
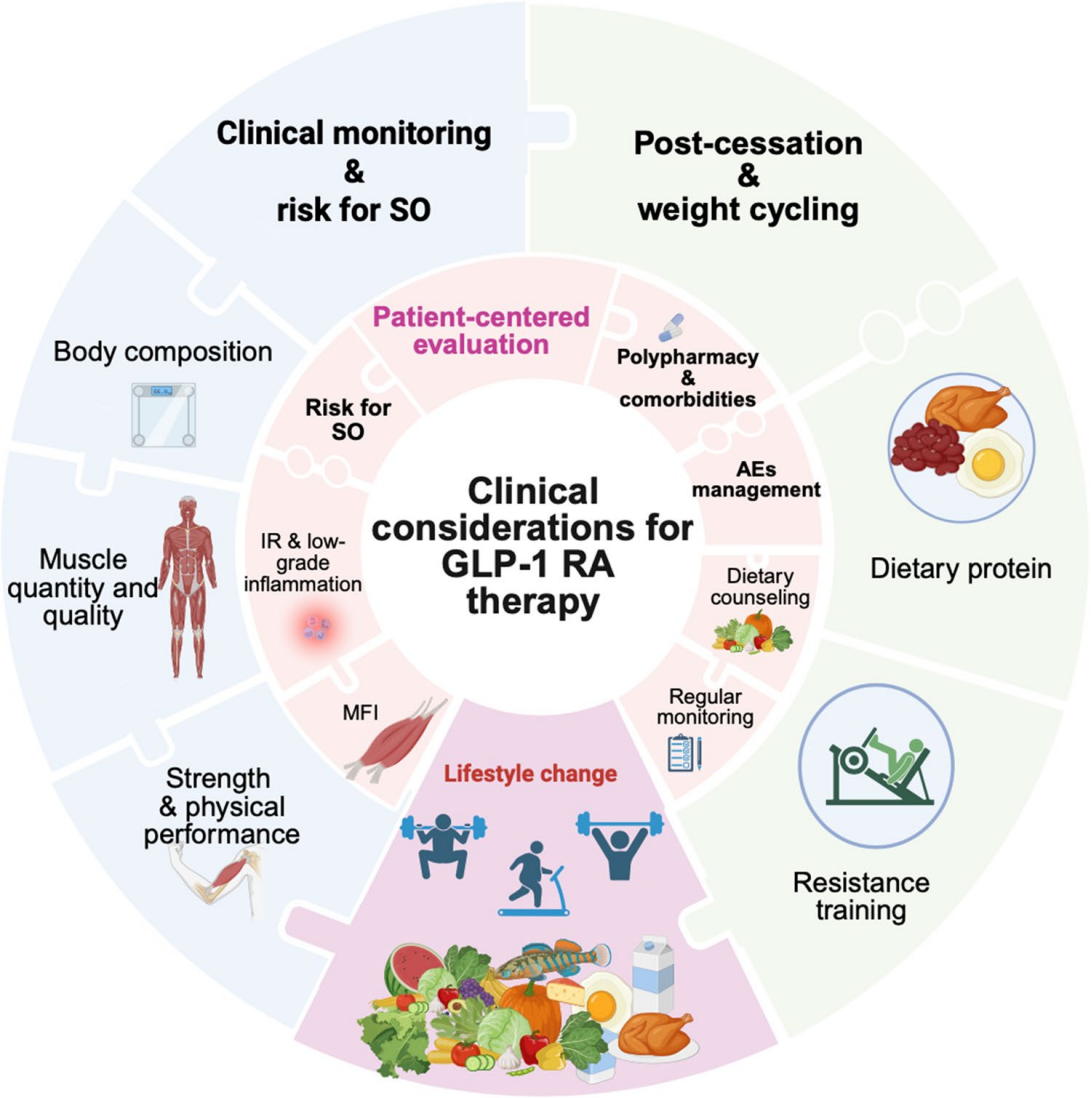
Schematic overview of the clinical management of GLP-1 RA therapy and the associated risk of SO. Abbreviations: AEs, adverse events; MFI, muscle fat infiltration; IR, insulin resistance; SO, sarcopenic obesity; GLP-1 RAs, GLP-1 receptor agonists. This figure is illustrated with BioRender.com

Weight regain is a common phenomenon after discontinuation of GLP-1 RAs, suggesting that the therapeutic benefits largely depend on treatment and subsequent management. Indeed, a recent meta-analysis reported that, while proportional to initial loss and varying by type of AOM, weight regain can range from 2.2 kg to 9.7 kg [101]. Furthermore, the meta-regression analysis conducted by Budini et al. showed that weight regain would likely stabilize at 75.6% (95% CI 68.5–82.7) of the weight initially lost during treatment, with approximately 40.2% of treatment-induced weight loss retained at one year post-discontinuation [102]. However, researchers have also highlighted a significant gap in the current literature regarding the precise body composition of the regained weight [102]. Cessation of GLP-1 RA therapy is associated with weight regain, and although current evidence does not provide conclusive findings regarding sarcopenia, the tendency to regain more fat than muscle mass, together with a potential decline in muscle quality, may pose a risk [98]. While data specific to GLP-1 RA discontinuation remain limited, a large-scale prospective study on weight cycling (mean age 63.6 years, at high risk of T2DM) reported that weight regain was typically characterized by an adverse shift in body composition, with FM returning to baseline levels while FFM declined by 1.50 kg (95% CI 0.66–2.35). Notably, even when initial weight gain was followed by subsequent weight loss, individuals retained a net increase in FM (1.70 kg), while FFM remained stable [103]. Given the complex and heterogeneous trajectory of FFM during weight regain and considering that FFM is a broader, less metabolically strategic compartment than specific lean mass, as previously discussed, evaluating the qualitative deterioration of muscle tissue emerges as a more critical objective. In this context, a 5-year post-semaglutide study reported that weight regain was associated with an increased MFI and reduced muscle quality [104]. Collectively, these findings highlight that post-cessation body composition changes extend beyond simple weight recovery, with potential implications for SO risk, particularly in older adults. These findings indicate that sustainable lifestyle approaches, such as diet and exercise, could help support the management of post-cessation weight regain, thereby helping to manage SO risk in older adults (Fig. 2).

GLP-1 RA therapy, when combined with tailored resistance exercise and CR with dietary counseling, may be effective in preserving muscle mass and function by optimizing body composition changes [84]. Adequate protein intake, both in quantity and quality, represents a critical component of preventive dietary strategies targeting sarcopenia [105, 106]. A minimum protein intake of 1 g/kg/day is recommended to maintain muscle mass in older adults [107]; moreover, several previous studies have shown that protein intake of 1.2–1.6 g/kg/day is associated with a lower risk of sarcopenia [106, 108].

Adequate protein intake may be difficult to achieve during GLP-1 RA therapy, as anorexia is one of the medication’s main mechanisms. Energy intake has been reported to decrease by 16–39% compared with similar patients not receiving GLP-1 RA treatment; however, dietary composition, particularly protein intake, is rarely reported in these studies [109]. The lack of detailed dietary assessment in many studies complicates the monitoring of sufficient protein intake; nonetheless, a few studies have examined dietary intake in detail. For instance, in a parallel-arm randomized controlled trial, 24-h dietary recall was used, and in the group receiving 14 weeks of CR combined with dietary counseling, the percentage of calories from dietary protein increased significantly compared with liraglutide monotherapy, whereas changes in total energy intake were similar between groups [110]. Similarly, in a cross-sectional study by Johnson et al., analysis of 3-day food records in individuals using GLP-1 RA for at least one month showed that 75% of participants reported having increased their protein intake after treatment, yet only 43% achieved the recommended intake of ≥ 1.2 g/kg/day; fiber, vegetable, and fruit intake remained inadequate, while saturated fat and sodium intake were high [111]. The results of both studies may reflect insufficient characterization of clinical practice, particularly regarding dietary approaches, and/or inadequate dietary counseling. Accordingly, the importance of tailored nutritional counseling by an RDN to optimize protein intake and preserve muscle mass and function during GLP-1 RA therapy should be emphasized. The development of GLP-1 RA-specific medical nutrition therapy (MNT) protocols, and the active involvement of an RDN in optimizing key dietary recommendations, including ensuring adequate protein intake (based on g/kg body weight rather than percentage of total energy), increasing fiber intake, and reducing saturated fat intake, is important.

Although GLP-1RA therapy facilitates mobility through weight loss, particularly in older adults, maintaining exercise and physical activity can be challenging due to side effects and fatigue; moreover, low mechanical loading may increase the risk of lean mass loss [84]. The protective effect of combining GLP-1 RA therapy with exercise has been previously reported, with Sandsdal et al. (2023) revealing reductions in abdominal obesity and inflammation, although muscle mass was not assessed [112]. Ongoing research is examining the effects of exercise, including resistance training in combination with GLP-1 RA therapy (ClinicalTrials.gov: NCT05786521, NCT04122716). Currently, resistance training is mainly considered a recommended approach for maintaining muscle mass [39].

## Conclusions

GLP-1 RAs offer remarkable opportunities for managing obesity-related comorbidities and disease burden, particularly in the aging population. However, potential risks and long-term uncertainties necessitate not only close monitoring in older adults but also a sustainable, lifestyle-centered management approach. Gradual dose escalation is crucial for mitigating AEs, while combination with other medications, including oral antidiabetic agents, and consideration of polypharmacy in older adults, as well as individualization of therapy, should consider comorbidities, gastrointestinal tolerability, and cost. Furthermore, the integration of telehealth, tele-nutrition, and digital platforms can enhance dietary counseling and patient monitoring across all stages of treatment. Future research is warranted to characterize the effects of GLP-1 RA therapy and post-cessation on SO risk in older adults, as well as to establish MNT protocols and define evidence-based clinical approaches tailored to this population. While the current body of evidence has predominantly focused on fat versus lean mass, relatively few studies (mostly subgroup analyses) have examined SMI, ASMI, and physical performance. Notably, older adults with obesity and preserved baseline muscle mass represent a particularly understudied phenotype, and dedicated studies are needed to determine whether this subgroup may differentially benefit from the muscle quality improvements associated with GLP-1 RA therapy. Further studies specifically addressing skeletal muscle quantity and quality, as well as functional capacity measures, with an emphasis on sarcopenia, are needed to elucidating effects of GLP-1 RAs on SO.

## Key References

- Žižka O, Haluzík M, Jude EB. Pharmacological Treatment of Obesity in Older Adults. Drugs & Aging. 2024 41(11):881–896. 10.1007/s40266-024-01150-9.
  - ○ This key reference addresses pharmacological obesity management through the lens of sarcopenia and SO, emphasizing the clinical management of nutritional and functional risks in the older population beyond weight loss.
- Linge J, Birkenfeld AL, Neeland IJ. Muscle Mass and Glucagon-Like Peptide-1 Receptor Agonists: Adaptive or Maladaptive Response to Weight Loss? Circulation. 2024, 150(16):1288–1298. 10.1161/CIRCULATIONAHA.124.067676.
  - ○ This review comprehensively addresses the effects of GLP-1 RA–mediated weight loss on muscle mass and quality in obesity management, highlighting the importance of assessing and preserving muscle health within a holistic approach.
- Karakasis P, Patoulias D, Fragakis N, Mantzoros CS. Effect of glucagon-like peptide-1 receptor agonists and co-agonists on body composition: Systematic review and network meta-analysis. Metabolism—Clinical and Experimental. 2025, 164: 156113. 10.1016/j.metabol.2024.156113.
  - ○ This meta-analysis assesses the effects of GLP-1 RAs on body composition across various components, providing a comprehensive overview of the effects associated with different agents and their applications.

## Data Availability

No datasets were generated or analysed during the current study.
